# A protocol for tactile function assessment using JVP domes: Feasibility study and preliminary results

**DOI:** 10.1002/brb3.3123

**Published:** 2023-06-19

**Authors:** Yung‐Jung Wang, Chung‐Tung Sung, Jian‐Jia Huang, Yu‐Cheng Pei, Bao‐Luen Chang

**Affiliations:** ^1^ School of Medicine, College of Medicine Chang Gung University Taoyuan Taiwan; ^2^ Department of Neurology Chang Gung Memorial Hospital at Linkou Medical Center Taoyuan Taiwan; ^3^ Department of Medical Education Chang Gung Memorial Hospital at Linkou Medical Center Taoyuan Taiwan; ^4^ Department of Physical Medicine and Rehabilitation Chang Gung Memorial Hospital at Linkou Medical Center Taoyuan Taiwan; ^5^ Center of Vascularized Tissue Allograft Chang Gung Memorial Hospital at Linkou Medical Center Taoyuan Taiwan; ^6^ Master of Science Degree Program in Innovation for Smart Medicine Chang Gung University Taoyuan Taiwan; ^7^ Neuroscience Research Center Chang Gung Memorial Hospital at Linkou Medical Center Taoyuan Taiwan

**Keywords:** grating orientation task, JVP dome, staircase method, tactile discrimination, tactile function

## Abstract

**Background:**

Touch is a crucial sense for perceiving the spatial characteristics of objects. The JVP dome was developed to evaluate tactile spatial acuity using a grating orientation task. There were few studies depicting sequences and details for the entire task, including practice, training, and testing sessions. Therefore, we proposed and elaborated a protocol for the grating orientation task using the staircase method, which required fewer testing trials compared with the method of constant stimuli.

**Methods:**

Twenty‐three healthy participants were enrolled in this experiment. The JVP domes with 11 different groove widths were used. Tactile discrimination thresholds were estimated using a two‐down‐one‐up staircase method. The experiment comprised practice, training, and testing sessions, conducted by trained examiners who performed grating stimulation on participants’ index fingerpads.

**Results:**

All participants passed the required accuracy in the practice and training sessions. Eight transition points were obtained in the testing session for each participant. The tactile discrimination thresholds were determined from the last six transition points. We obtained the mean tactile discrimination threshold as 1.8 ± 0.75 mm (*n* = 23). The results demonstrated that the proposed protocol was successfully applied to assess tactile discrimination thresholds.

**Conclusions:**

The present study investigated the protocol of grating orientation tasks requiring a small number of testing trials with the assurance of the task quality. The feasibility study and preliminary results indicated the potentiality of this protocol for future clinical application.

## Graphical abstract

1.Touch is a crucial sense for perceiving the spatial characteristics of objects. The JVP dome was developed to evaluate tactile spatial acuity using a grating orientation task. 2. In this study, we proposed and elaborated a protocol for the grating orientation task using a two‐down‐one‐up staircase method to estimate tactile discrimination thresholds. The protocol comprised practice, training, and testing sessions, conducted by trained examiners who performed grating stimulation on participants’ index fingerpads. 3. The protocol successfully reduced the number of testing trials while ensuring task quality, highlighting its potential for future clinical applications.

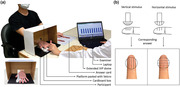

## 1 INTRODUCTION

Touch is a crucial sense for perceiving the spatial characteristics of objects. The traditional measurement of a two‐point discrimination threshold has been widely employed for assessing tactile spatial acuity. However, a major drawback of this method is its inability to control the influence of nonspatial cues (Johnson & Phillips, 1981; Tong et al., 2013). In response to this limitation, a novel measurement was developed to assess tactile spatial sensitivity using grating domes—the JVP dome. The JVP dome was introduced by Johnson, Van Boven, and Phillips in 1981 to assess grating orientation discrimination capacity (Johnson & Phillips, 1981). JVP domes have different grating widths with the original grooves ranging from 0.35 to 3 mm (Sathian & Zangaladze, 1996; Wohlert, 1996), which present different challenges to spatial processing. The JVP dome is used to evaluate an individual's ability to identify the orientation of grating presented to the skin in a two‐alternative forced‐choice design, providing a more consistent, reliable, and relatively cheap measurement to quantify human tactile capacity for spatial resolution (Holmes & Tamè, 2023; Van Boven & Johnson, 1994a).

Since the JVP dome was introduced, the need for normative data has been raised. Studies of the grating orientation task have demonstrated that tactile thresholds differ among body parts and decline from the index to the middle finger and from the middle to the ring finger but do not differ between the right and left hands (Craig & Lyle, 2001; French et al., 2022; Sathian & Zangaladze, 1996; Van Boven & Johnson, 1994a; Vega‐Bermudez & Johnson, 2001). Furthermore, spatial discrimination, especially at the fingerpad, markedly declines as age increases (Stevens & Choo, 1996; Stevens & Patterson, 1995; Wohlert, 1996). Researchers have not only used the JVP dome to characterize tactile function in healthy individuals but also in individuals with diseases. For example, performance in grating orientation discrimination corresponded consistently to disease progression in patients with peripheral nerve injury (Van Boven & Johnson, 1994b) and was thus considered a valid and precise measurement for evaluating sensory function after nerve repair (Klein et al., 2016). The grating orientation task is regarded as a standard method for assessing tactile acuity.

The grating orientation task has been applied in various somatosensory studies; however, there were few studies depicting sequences and details for the entire experimental procedure, including practice, training, and testing sessions. Tremblay et al. (2003) carried out the experiment with ten practice trials and proceeded with formal testing by presenting grating domes in a sequence of increasing difficulties, namely, the method of constant stimuli. Holmes and Luigi (2023) outlined a training procedure using the 3 mm‐dome and estimated the range of grating orientation thresholds prior to the formal testing session, which was also performed using the method of constant stimuli. Studies of Vega‐Bermudez and Johnson (2001) and Wong et al. (2011) used staircase method (Levitt, 1971; Wetherill & Levitt, 1965) in the testing session, which was characterized by requiring less testing trials, having flexibility to cover a wide range of participant sensitivities, and showing equal difficulty perceived by different participants (Grant et al., 2006; Holmes & Tamè, 2023), but the practice and training sessions before testing were not described. Considering the further applications in clinical conditions, the staircase method may provide the advantage to reduce testing trials with similar accuracy (Grant et al., 2006). Therefore, an elaborate protocol of the grating orientation task method is necessary.

In this pilot study, we proposed a modified protocol based on the study of Vega‐Bermudez and Johnson (2001) and investigated it in detail, including a practice session, a training session, and a testing session, using two‐down‐one‐up staircase method. We expected that this protocol would yield an accurate quantification for tactile function assessment.

## 2 MATERIALS AND METHODS

### 2.1 Participants

Twenty‐three healthy adult participants (13 men and 10 women, aged between 40 and 65 years) were recruited. All participants reported normal tactile sensation and denied systemic or neurological diseases. Written informed consent was obtained from participants. The exclusion criteria were: (1) sensory impairment (anesthesia or hypoesthesia) or sensation change (paresthesia or hyperesthesia) in testing hand; (2) a lesion in peripheral or central nervous systems in testing hand; (3) alcoholism or history of alcoholism; (4) rheumatoid disease; (5) complex regional pain syndrome; (6) hypothyroidism; (7) fibromyalgia; (8) end‐stage renal disease; and (9) inability to follow the experiment's instructions. The study protocol and procedure were approved by the Institutional Review Board of the Chang Gung Medical Foundation (IRB No.: 202001628B0A3). All methods were performed according to the regulations of the Human Subjects Research Act in Taiwan and the Declaration of Helsinki (1975). The study and procedure details were explained clearly to participants.

### 2.2 Examiner training

The examiners were trained before performing the experiment. The indentation time for JVP dome application to skin was standardized to 1 s, with moderate force resulting in approximately 2 mm of skin displacement (Van Boven et al., 2000). The experimenters practiced this action hundreds of times using a timer to ensure that the entire procedure could be undertaken with approximately equal indentation times.

### 2.3 Experimental setup

#### 2.3.1 JVP domes

The diameter of each dome was 20 mm with a circular, convex grating surface. The domes, with equidistant grooves and bars, were mounted on a cylindrical handle. The JVP domes chosen in our experiment comprised 11 domes with different groove widths (5, 4, 3, 2.5, 2, 1.5, 1.25, 1, 0.75, 0.5, and 0.35 mm). In comparison to the original JVP set, modifications were made by incorporating 5‐ and 4‐mm domes based on the study by Vega‐Bermudez and Johnson (2001) involving middle‐aged participants. Additionally, a 2.5‐mm dome was added to address the significant decrease in discrimination performance resulting from the abrupt transition from the 3‐ to 2‐mm dome (Remblay et al., 2000; Tremblay et al., 2003).

#### 2.3.2 Experimental environment and instructions for the two‐alternative forced‐choice test of tactile orientation

**FIGURE 1:**
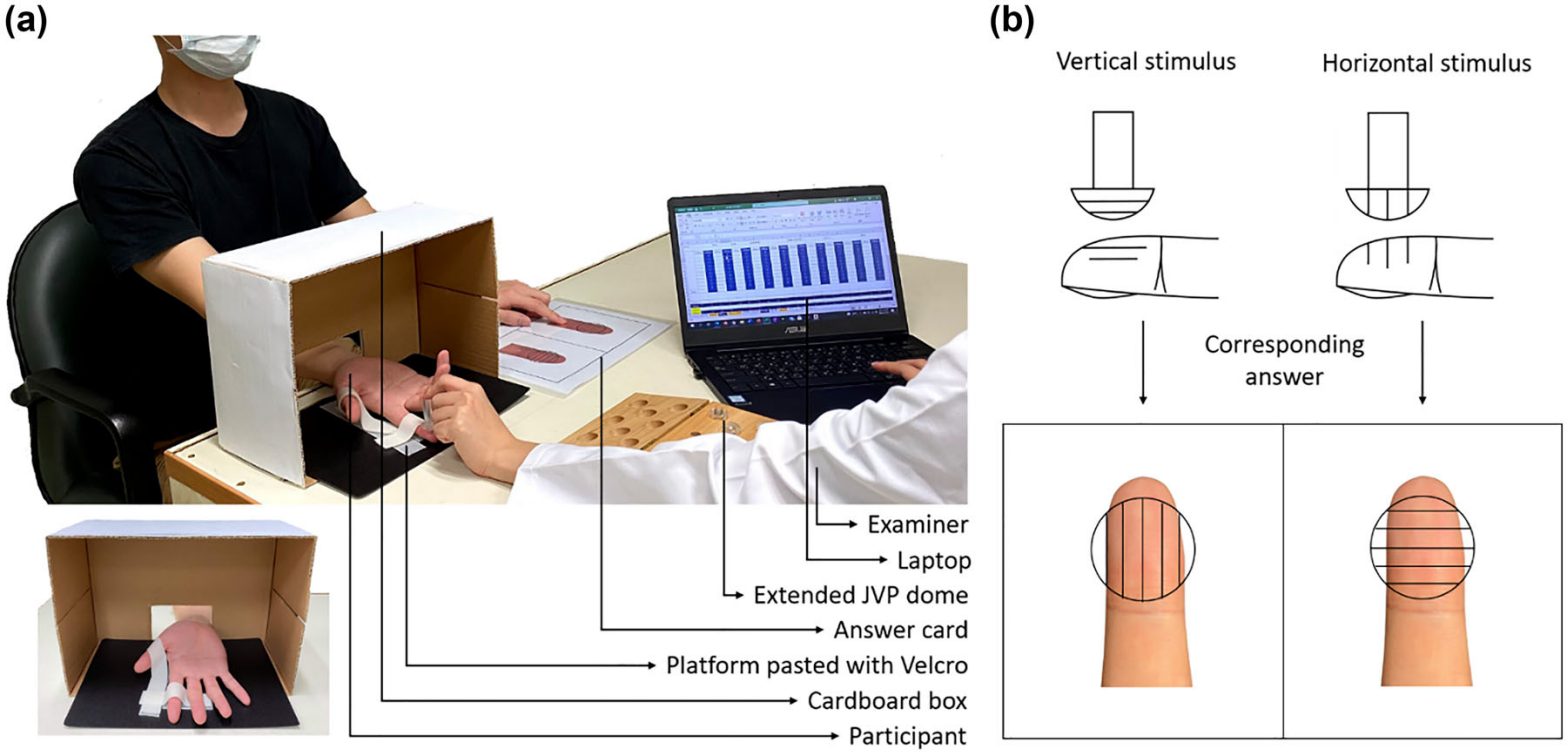
**Experimental setup and answer mode**. (a) The participant sat in front of the examiner and maintained a palm‐up position with the testing hand inserted through the square opening of the cardboard box. The proximal phalanx of the thumb and middle phalanx of the index finger were immobilized using Velcro as shown in the bottom left of the figure. The answer card was placed on the non‐testing hand side. The answers were recorded by the examiner using a laptop. (b) The illustrations on the answer card by which participants reported answers corresponding to the perceived stimulation at the testing finger. If a vertical stimulus was perceived, the participants placed the non‐testing hand on the side of the vertical illustration on the answer card, and vice versa.

The experimental environment setup is shown in Figure 1a. A cardboard box (30 × 22 × 15 cm^3^) with a square opening (11 × 7 cm^2^) for placing the testing hand and to block the view of the stimulus was set in front of the participant. A platform with Velcro was placed inside the box for the fixation of the testing hand. The testing hand was fixed to the platform palm‐side up at the proximal phalanx of the thumb and middle phalanx of the index finger using Velcro. An answer card was placed on the side of the non‐testing hand, printed with two illustrations depicting vertical and horizontal schematics on the left and right sides, respectively.

**FIGURE 2:**
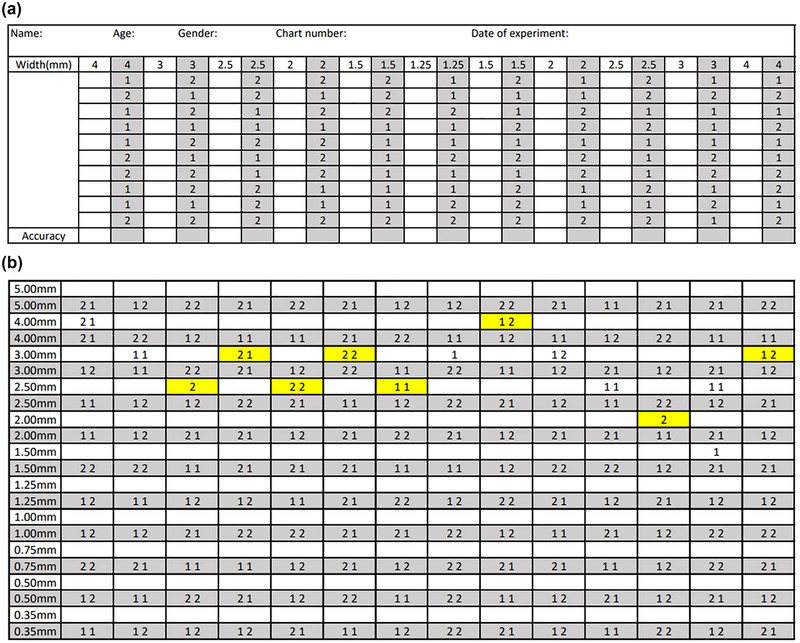
**Answer record tables for training and testing sessions**. (a) Blank training session table. The table shows randomized numbers (1 or 2) instructing the examiner on the orientation of the stimulus. Number 1 indicated vertical stimulus and Number 2 indicated horizontal stimulus. The blank areas next to the randomized numbers were for recording corresponding answers. (b) A testing session record table showing a sample result using the staircase method. The stimulation started with the 4‐mm dome and followed the two‐down‐one‐up rule. For example, if the participant incorrectly responded to 2.5‐mm dome stimulation once, the following stimulation would use the 3‐mm dome; if the participant correctly responded to 3‐mm dome stimulation twice, the following stimulation would use the 2.5‐mm dome. The yellow areas denote the eight transition points.

Tactile stimulations using the JVP domes were performed by an examiner with either vertical (the gratings aligned parallel to the long axis of the finger) or horizontal (the gratings aligned transverse to the long axis of the finger) orientation. Each single stimulus was presented once on the participant's index fingerpad for approximately 1 s. The sequence of tactile stimulation was randomly assigned using balanced orientations. A two‐alternative forced‐choice test was applied in which the participant reported perceived orientation for each stimulation. The participant did so using the non‐testing hand to point to either the vertical or horizontal illustration on the answer card. If vertical orientation was perceived, the participant placed the non‐testing hand on the vertical illustration side of the answer card, and vice versa (Figure 1b). Answers were recorded digitally by the examiner. In the answer record tables, vertical and horizontal orientations were denoted as “1” and “2,” respectively (Figure 2).

### 2.4 Experimental procedures

**FIGURE 3:**
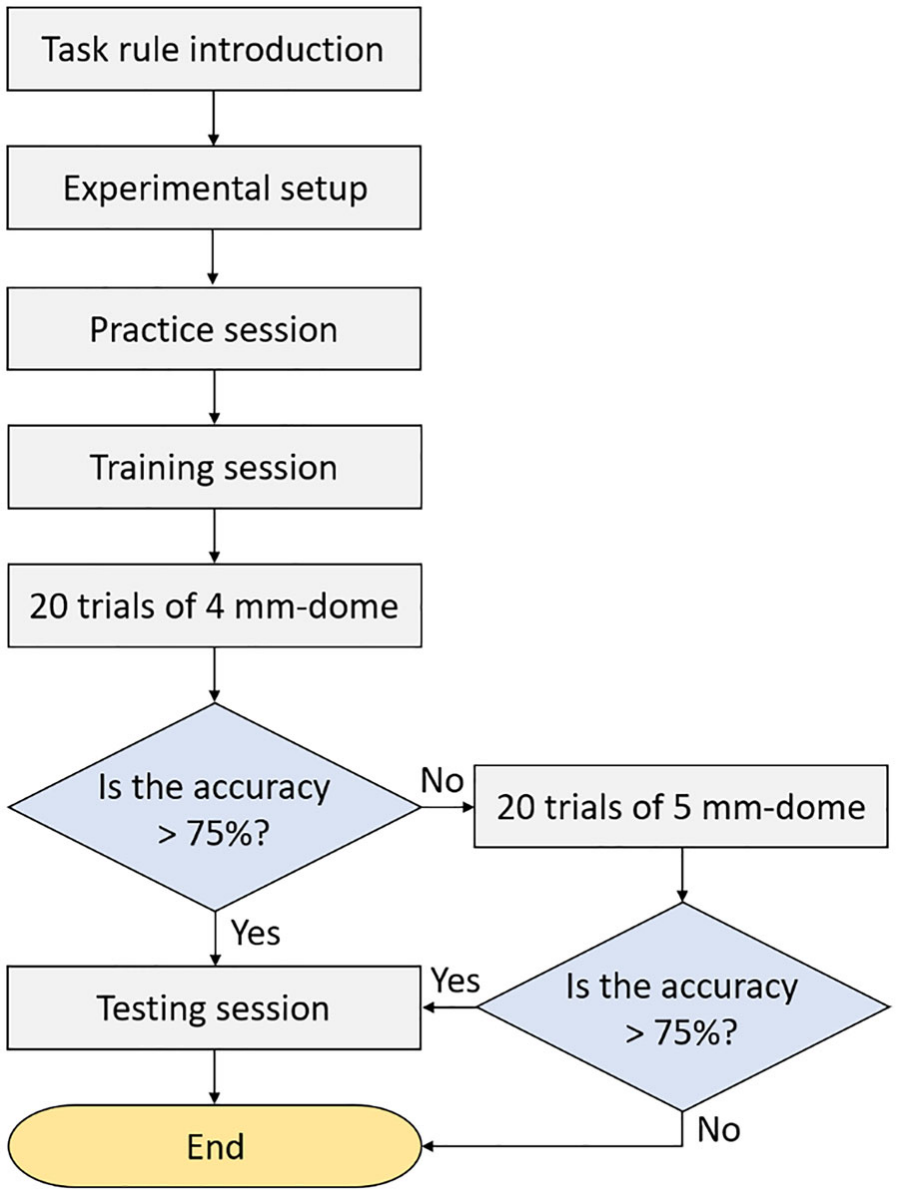
**Flowchart of the experimental procedure**.

The experiment comprised practice, training, and testing sessions, with a 5‐min break between training and testing sessions, lasting approximately 20–30 min in total (Figure 3).

Before each session began, we explained the rules of the task and instructed the participants to place their testing hands palm‐side up and their non‐testing hands on the answer card.

#### 2.4.1 Practice session

The 4‐mm dome was used for the practice session since most healthy adults could discriminate it easily (Tremblay et al., 2003).

- First, practice was conducted without the use of the cardboard box for the participants to observe the orientation of the JVP dome during stimulation. After each stimulation on the fingerpad, the participants reported the answer. This practice session concluded after three consecutive correct answers.
- Second, practice was conducted using the cardboard box placed over participants’ hands to block their view during stimulation (Figure 1a). Before each stimulation, the orientation of the JVP dome was observed by the participants. This practice session concluded after three consecutive correct answers.

#### 2.4.2 Training session

- For the training session, the setup used was the same as that in step b of the practice session (Figure 1a), except that the participants could not first observe the orientation of the JVP dome. Stimulation was conducted with grating widths from 4 to 1.25 mm and then in reverse with grating widths from 1.5 to 4 mm. Stimulation with each dome was conducted 10 times before advancing to the next. A total of 110 trials were conducted.
- After the training process, 20 trials using the 4‐mm dome were conducted to evaluate whether the participants were well‐trained. If the discrimination accuracy was higher than 75%, the participants were considered to have successfully completed the training session. If the discrimination accuracy was below 75%, indicating that the participants might not be familiar enough with the protocol or that their tactile function might be suboptimal, the participants then completed 20 trials using the 5‐mm dome. If discrimination accuracy using the 5‐mm dome was higher than 75%, the participants were considered to have successfully completed the training session. If discrimination accuracy remained below 75%, the task was ended, and the tactile discrimination threshold of the participant was recorded as 5 mm.

#### 2.4.3 Testing session

- In the testing session, stimulation was conducted using a modified two‐down‐one‐up staircase method starting with the 4‐mm dome. Namely, if the participant gave two consecutive correct answers, the next lowest grating width would be used; if the participant gave one incorrect answer, the next greatest grating width would be used (Figure 2b).
- A transition point was identified where the stimulation changed from decreasing widths to increasing widths and vice versa. The examiner recorded these points digitally. The session was completed when eight transition points were identified.

#### 2.4.4 Data analysis and statistics

The average of the grating widths at the last six transition points was used to determine the tactile discrimination threshold, which corresponds to 70.71% correct performance (Fujii & Schlaug, 2013; Grant et al., 2006; Kühner et al., 2014; McCourt & Paulson, 1994; Stevens & Choo, 1996; Vega‐Bermudez & Johnson, 2001; Wong et al., 2011). All data were presented as mean ± standard deviation.

## 3 RESULTS

The participants enrolled in this study were all right‐hand dominant, as confirmed using the Edinburgh Handedness Inventory. Four were tested using the left hand due to carpal tunnel syndrome or previous injury in the right hand. The highest education level was junior high school for two participants, senior high school for three participants, and university for the remaining participants (Table 1). All participants completed the entire task within 30 min without any adverse events occurring.

**TABLE 1:** Participant characteristics.

*Participant characteristics*					
Participant	Age	Gender	Dominant hand^a^	Testing hand	Education level
1	54	Female	Right	Right	University
2	49	Female	Right	Left^b^	Junior high
3	55	Female	Right	Right	University
4	48	Male	Right	Right	University
5	47	Male	Right	Right	University
6	47	Male	Right	Right	University
7	53	Male	Right	Right	University
8	48	Female	Right	Left^b^	Senior high
9	52	Female	Right	Right	Senior high
10	50	Male	Right	Right	University
11	52	Male	Right	Left^b^	University
12	50	Female	Right	Right	University
13	54	Female	Right	Right	Senior high
14	59	Male	Right	Left^b^	Junior high
15	51	Male	Right	Right	University
16	40	Male	Right	Right	University
17	46	Male	Right	Right	University
18	41	Male	Right	Right	University
19	43	Female	Right	Right	University
20	49	Male	Right	Right	University
21	45	Female	Right	Right	University
22	49	Female	Right	Right	University
23	59	Male	Right	Right	University

^a^ Participants’ dominant hands were confirmed using the Edinburgh Handedness Inventory.

^b^ The grating orientation task was completed using the left index finger due to carpal tunnel syndrome or previous injury in the right hand for these participants.

First, we examined whether the participants correctly understood our instructions. All participants fully understood the instructions and correctly determined the orientations of the stimulation they received in the practice session (Table 2), indicating the use of clear instructions.

**TABLE 2:** Performance in the practice session.

Participant		Practice without the box			Practice with the box		
	Stimulus	1	2	2	1	2	1
1–23	Response	1	2	2	1	2	1

*Note*: The numbers (1: vertical orientation, 2: horizontal orientation) in the top row indicate the orientation of stimulation and the following row depicts the answers given by the 23 participants for the corresponding stimuli. All participants correctly responded to all stimuli during practice both with and without the box.

**FIGURE 4:**
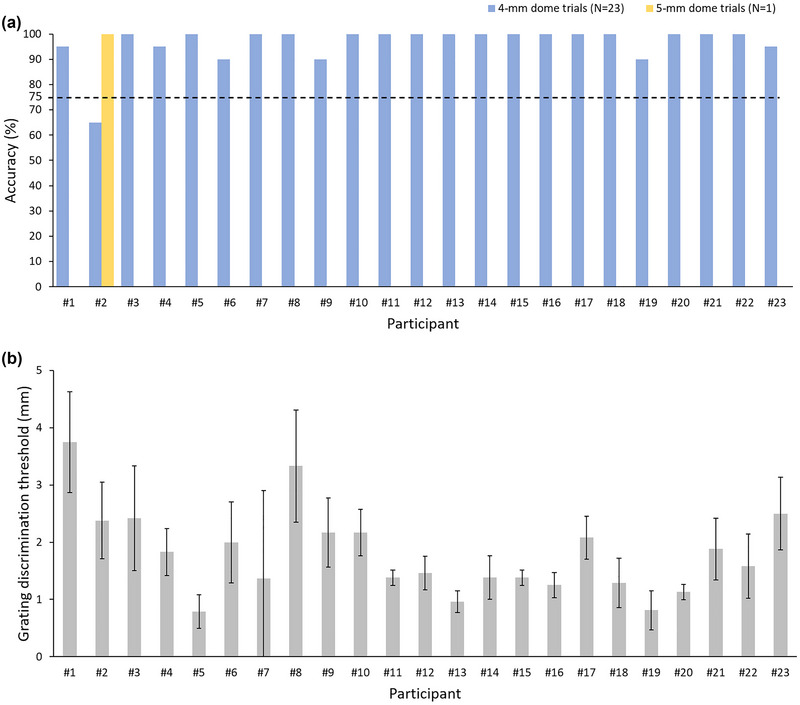
**Training and testing session performance**. (a) Answer accuracy in the training session. The accuracy of 22 of the 23 participants was above 75% in 20 trials using the 4‐mm dome. Although participant 2 exhibited only 65% accuracy in the 4‐mm dome trials, she exhibited 100% accuracy in 5‐mm dome trials. (b) Tactile discrimination thresholds obtained from the testing session. Data are presented as mean ± standard deviation (SD).

Second, we verified the feasibility of the training session protocol by examining whether participants could complete the session with the required accuracy. The results showed that 22 of the 23 participants exhibited an accuracy of greater than 75% in 20 trials using the 4‐mm dome, and participant 2 exhibited an accuracy of greater than 75% in 20 trials using the 5‐mm dome (Figure 4a). All participants completed the training session in 20 trials using either the 4‐ or 5‐mm dome, indicating that the training protocol adequately allowed participants to become familiar with the testing protocol.

Third, we examined the testing session protocol. We assessed whether tactile discrimination thresholds could be determined using the staircase method and whether the JVP domes used were adequate. Eight transition points were obtained for each participant using the testing protocol, and the tactile discrimination threshold was determined by the last six transition points. The mean number of trials was 28.83 ± 5.16. The mean tactile discrimination threshold for the 23 participants was 1.8 ± 0.75 mm (Figure 4b). The thresholds of all participants were within the range of grating widths, indicating that, for these healthy middle‐aged participants, tactile discrimination thresholds could be measured using the selected grating width range.

## 4 DISCUSSION

This study demonstrated that, first, all participants could achieve the required accuracy in the practice and training sessions by following the instructions. Second, in the testing session, the tactile discrimination thresholds of all participants could be obtained using the staircase method, demonstrating the applicability of the protocol using the JVP domes. Third, the entire assessment was completed within 30 min, indicating its feasibility. Fourth, no discomfort or adverse events were reported during the experiments, indicating its safety and tolerability.

The usage of the 4‐mm dome in the practice session, where the participants were able to observe the orientation prior to receiving stimulation, effectively prevented inadequate comprehension during the formal task. In addition, the practice and training sessions excluded individuals with compromised tactile function. In instances where certain participants were unable to complete the task due to either an inability to perceive the stimulus or comprehend the task (Holmes & Tamè, 2023), the implementation of adequate practice and training in our protocol play a crucial role in ensuring the quality of the subsequent formal testing session.

The proposed staircase method utilized in the present study offered a significant advantage by effectively reducing the number of required trials, thus benefiting its application in clinical purpose. Specifically, the mean number of trials in this method was 28.83 ± 5.16, which is notably lower than the hundreds of trials typically required by the method of constant stimuli (Grant et al., 2006; Tremblay et al., 2003; Van Boven et al., 2000).

Our study showed that the grating widths of the JVP domes use in our protocol were satisfactory. During the formal task, 17.4% and 43.5% of the participants required the 5‐ and 4‐mm domes, respectively, highlighting the necessity of incorporating these particular domes. Furthermore, tactile function declines with age (Stevens & Choo, 1996; Wohlert, 1996). The mean threshold of spatial acuity in adults aged 55–86 years is twice that in adults aged 21–26 (2.5 vs. 1.2 mm) (Manning & Tremblay, 2006) and is 1.23 mm in adults with mean age 35‐year old (Vega‐Bermudez & Johnson, 2001). In this study, the mean tactile discrimination threshold was 1.80 mm in participants aged 40–65 years (mean age 50‐year old). The grid widths applied in this study were suitable for the discrimination thresholds of both young and older adults, indicating the benefits of our JVP domes.

The inter‐examiner reliability of the protocol was critical. First, Bleyenheuft and Thonnard (2007) validated that variance in pressing force applied to JVP domes among examiners does not affect estimated threshold (Gibson & Craig, 2006). Second, although the examiners were trained before conducting the experiment, the perfect orientation of the domes when presented to the fingerpad could not be guaranteed. However, because only two orientations could be indicated by participants (vertical and horizontal), we hypothesize that slight deviations in orientation did not affect the participants’ judgment of orientation. Building upon similar methods that enable more consistent stimulations using autonomic apparatuses (French et al., 2022; Goldreich et al., 2009; Pei et al., 2014), our team is currently engaged in the development of a robotic instrument. This instrument aims to achieve precise and reliable stimulus orientation while minimizing the occurrence of operational errors and preventing examiner fatigue.

**FIGURE 5:**
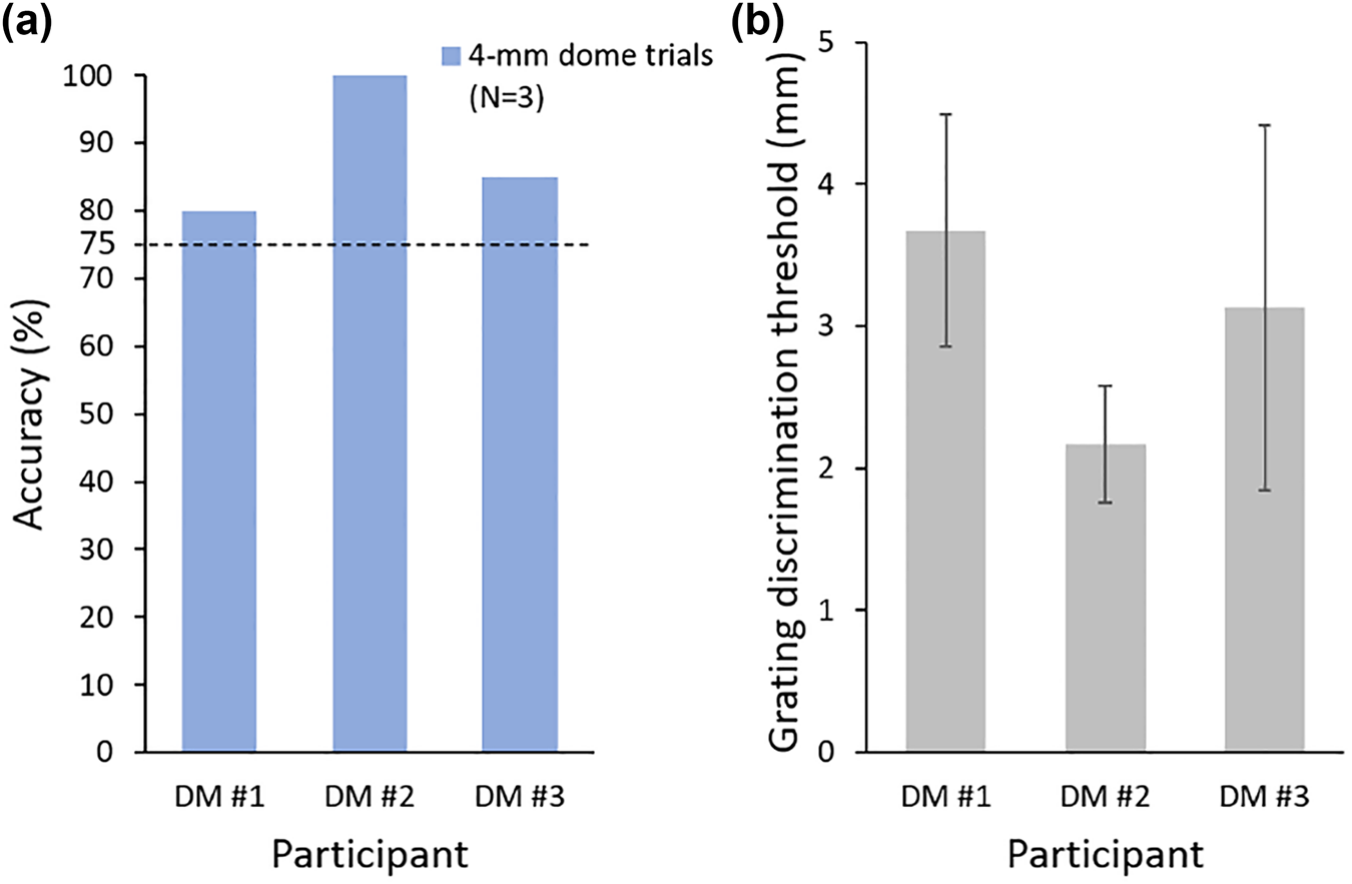
**Training and testing session performance of patients with diabetes mellitus (DM)**. (a) Answer accuracy in the training session. All three diabetic patients passed the 4‐mm dome trials with accuracy above 75%. (b) Tactile discrimination thresholds obtained from the testing session. Data are presented as mean ± standard deviation (SD).

We further attempted to apply this protocol to a small sample of patients with diabetes mellitus to investigate the alterations in hand tactile function resulting from peripheral neuropathy. The three middle‐aged diabetic patients were engaged and were able to complete the entire procedure without any difficulties. Their grating discrimination thresholds were 3.67, 2.17, and 3.13 mm, respectively (as shown in Figure 5). This result provides further support for the potential clinical application of the protocol.

This study has several limitations: (1) ensuring the consistent manual presentation of the same orientation for each trial was challenging, and (2) the potential limitations in generalizing our findings to patients with advanced age, neuropathy, or stroke. However, our long‐term goal is to apply this protocol to clinical tactile function assessment, such as in screening for peripheral neuropathies, evaluating sensory recovery following nerve reconstruction, and assessing sensory impairment caused by central nervous diseases.

## 5 CONCLUSION

This study introduced a modified tactile function assessment using JVP domes with staircase method and examined the grating orientation task in both practice and training sessions. The protocol successfully reduced the number of testing trials while ensuring task quality, highlighting its potential for future clinical applications.

## AUTHOR CONTRIBUTIONS

Yung‐Jung Wang, Chung‐Tung Sung, Jian‐Jia Huang, Yu‐Cheng Pei, and Bao‐Luen Chang designed the experiment. Yung‐Jung Wang, Chung‐Tung Sung, Jian‐Jia Huang, Yu‐Cheng Pei, and Bao‐Luen Chang performed the experiment. Yung‐Jung Wang, Chung‐Tung Sung, Jian‐Jia Huang, Yu‐Cheng Pei, and Bao‐Luen Chang analyzed the data. Yung‐Jung Wang, Chung‐Tung Sung, Jian‐Jia Huang, Yu‐Cheng Pei, and Bao‐Luen Chang wrote the manuscript. All authors interpreted the results of experiments. All authors edited, revised, and approved the final version of the manuscript.

## CONFLICT OF INTEREST STATEMENT

All authors have no conflict of interests to declare.

### PEER REVIEW

The peer review history for this article is available at https://publons.com/publon/10.1002/brb3.3123.

## Data Availability

The data that support the findings of this study are made openly and available in this published article.
